# ACUTE EFFECTS OF EDIBLE CANNABIS FOR OLDER ADULTS: THE GOOD, THE BAD, AND THE NEUTRAL

**DOI:** 10.1093/geroni/igae098.1242

**Published:** 2024-12-31

**Authors:** Angela Bryan, Carillon Skrzynski, Madeline Stanger, Gregory Giordano, Dana Lervick, Tatum Broyhill, Brian Tracy

**Affiliations:** University of Colorado Boulder, Boulder, Colorado, United States; University of Colorado Boulder, Boulder, Colorado, United States; University of Colorado Boulder, Boulder, Colorado, United States; University of Colorado Boulder, Boulder, Colorado, United States; Colorado State University, Fort Collins, Colorado, United States; Colorado State University, Fort Collins, Colorado, United States

## Abstract

Many older adults use cannabis to alleviate pain, increase sleep quality, and mitigate symptoms of anxiety and/or depression. Yet few studies assess co-occurring effects of cannabis use that may be more salient to older adults. We recruited 234 participants (average age 70.46 (sd=5.83) who either did (n=175) or did not (n=59) want to use edible cannabis for pain, sleep disturbance or negative affect. We compared acute effects on balance, mood, and memory before and after consumption of edible cannabis products differing in cannabinoid ratio (CBD dominant, THC dominant, CBD+THC). Among older adults who used cannabis, we observed balance impairment from pre to 60-minutes post use (p<.01). Those who used cannabis experienced improvement in pain from pre to 60-minutes post use (p=.001) and higher concentrations of THC in blood (measured at time of use) were associated with greater decreases in pain (p<.05). Older adults who used cannabis also experienced increases in subjective intoxication (p<.001); higher THC in blood was associated with greater intoxication (p<.001) while higher CBD in blood was associated with lower intoxication (p<.05). There were few significant differences between those who did versus did not use cannabis on memory and cognitive tasks. We add to the growing literature examining cannabis use among older persons and identify significant outcomes experienced by those who use edible cannabis products. Disseminating this work among clinicians may facilitate more informed conversations with older persons who may consider cannabis as a therapeutic alternative.

